# 1,4,5,8-Tetra­isopropyl­anthracene

**DOI:** 10.1107/S1600536810030837

**Published:** 2010-08-11

**Authors:** Chitoshi Kitamura, Hideki Tsukuda, Takeshi Kawase, Takashi Kobayashi, Hiroyoshi Naito

**Affiliations:** a Department of Materials Science and Chemistry, Graduate School of Engineering, University of Hyogo, 2167 Shosha, Himeji, Hyogo 671-2280, Japan; b Department of Physics and Electronics, Graduate School of Engineering, Osaka Prefecture University, 1-1 Gakuencho, Naka-ku, Sakai, Osaka 599-8531, Japan

## Abstract

The mol­ecules of the title compound, C_26_H_34_, possess crystallographically imposed inversion symmetry. The anthracene ring system is planar within 0.038 (1) Å. The two methyl groups in each independent isopropyl group are oriented on either side of the anthracene plane. In the crystal structure, the mol­ecules adopt a herringbone-like arrangement without π–π stacking.

## Related literature

For the preparation and solid-state fluorescence studies of 1,4,5,8- tetra­alkyl­anthracenes, see: Kitamura *et al.* (2007 ▶). For a related structure, see: Kitamura *et al.* (2010 ▶). For related herringbone structures, see: Curtis *et al.* (2004 ▶).
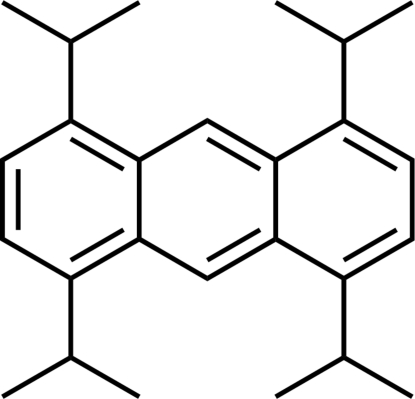


## Experimental

### 

#### Crystal data

C_26_H_34_
*M* *_r_* = 346.53
Monoclinic, 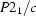

*a* = 6.546 (3) Å
*b* = 10.357 (5) Å
*c* = 15.808 (8) Å
β = 98.289 (8)°
*V* = 1060.5 (9) Å^3^
*Z* = 2
Mo *K*α radiation
μ = 0.06 mm^−1^
*T* = 223 K
0.50 × 0.07 × 0.05 mm

#### Data collection

Rigaku/MSC Mercury CCD area-detector diffractometer
Absorption correction: numerical (*NUMABS*; Higashi, 2000 ▶) *T*_min_ = 0.991, *T*_max_ = 0.996
9107 measured reflections
2817 independent reflections
1921 reflections with *I* > 2σ(*I*)
*R*_int_ = 0.043

#### Refinement

*R*[*F* ^2^ > 2σ(*F* ^2^)] = 0.063
*wR*(*F* ^2^) = 0.180
*S* = 1.07
2817 reflections
122 parameters
H-atom parameters constrained
Δρ_max_ = 0.28 e Å^−3^
Δρ_min_ = −0.24 e Å^−3^

### 

Data collection: *CrystalClear* (Rigaku/MSC, 2006 ▶); cell refinement: *CrystalClear*; data reduction: *CrystalClear*; program(s) used to solve structure: *SIR2004* (Burla *et al.*, 2005 ▶); program(s) used to refine structure: *SHELXL97* (Sheldrick, 2008 ▶); molecular graphics: *ORTEP-3 for Windows* (Farrugia, 1997 ▶); software used to prepare material for publication: *WinGX* (Farrugia, 1999 ▶).

## Supplementary Material

Crystal structure: contains datablocks global, I. DOI: 10.1107/S1600536810030837/ci5142sup1.cif

Structure factors: contains datablocks I. DOI: 10.1107/S1600536810030837/ci5142Isup2.hkl

Additional supplementary materials:  crystallographic information; 3D view; checkCIF report

## Figures and Tables

**Fig. 1.**
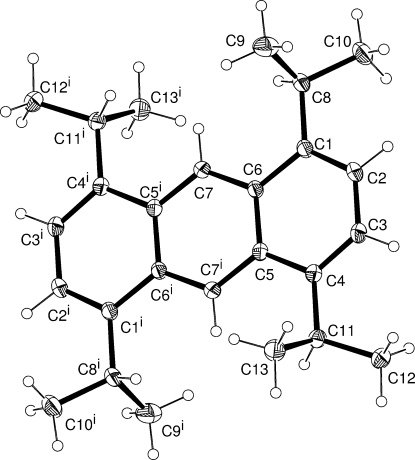
The molecular structure of (I), showing the atomic numbering numbering and 30% probability displacement ellipsoids for non-H atoms. Symmetry code: (i) 1 -x, -y, -z.

**Fig. 2.**
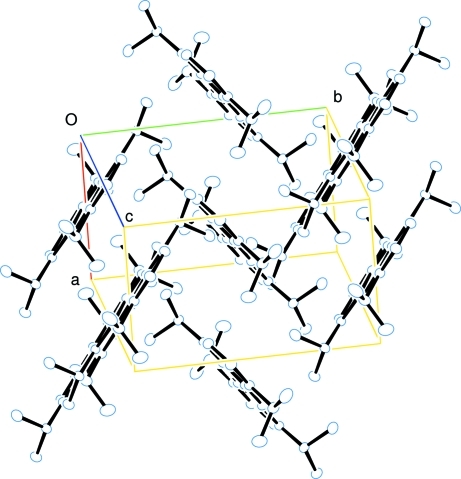
The packing diagram of (I). Hydrogen atoms have been omitted for clarity.

## supplementary crystallographic information

### Comment

Solid-state packing effects play an important role in the performance of electronic and photonic materials (Curtis *et al.*, 2004). However, there has been relatively little research on the correlation between solid-state packing patterns and fluorescence properties. Further, molecular design to control solid-state fluorescence is not fully understood. We have recently found that the introduction of linear alkyl side chains onto anthracene nucleus at the 1, 4, 5 and 8 positions brought about drastic changes in alkyl conformation, packing pattern, and solid-state fluorescence (Kitamura *et al.*, 2007). To investigate the effects of branched alkyl side chains, we embarked on the investigation on 1,4,5,8-tetraisoalkylanthracenes. Herein we report the X-ray analysis of the title compound (I).

The molecular structure of (I) is shown in Fig. 1. The molecule possesses a center of inversion, and half of the formula unit is crystallographically independent. The anthracene unit is essentially planar. The molecular structure is similar to that of 1,4,7,10-tetraisopropyltetracene (Kitamura *et al.*, 2010). Two terminal methyl groups of the two isopropyl groups at the 1 and 4 positions point upward, and two methyl groups of the other two isopropyl groups at 5 and 8 positions point downward. Thus, the torsion angles C6—C1—C8—C9 and C3—C4—C11—C13 are 80.72 (18) and 107.76 (16)°, respectively. Another two terminal methyl groups of the two isopropyl groups are nearly coplanar with the anthracene plane, and the C2—C1—C8—C10 and C3—C4—C11—C12 torsion angles are 25.6 (2) and -15.2 (2)°, respectively. In the crystal, the molecules adopt a herringbone-like (two-dimensional) arrangement as shown in Fig. 2. There are no π-π interatcions along the stacking direction.

To examine the influence of crystal packing on the solid-state fluorescence properties, the fluorescence spectrum and the absolute quantum yield of (I) were measured by a Hamamatsu Photonics PMA11 calibrated optical multichannel analyzer with a solid-state blue laser (*λ*_ex_ = 377 nm) and a Labsphere IS-040-SF integrating sphere, respectively. Crystals of (I) exhibited a structured fluorescence spectrum with fluorescence maxima at 432 and 450 nm. The quantum yield of crystals of (I) was very high (*Φ* = 0.80). Among 1,4,5,8-tetraalkylanthracenes, the *n*-propyl derivative had the largest quantum yield of 0.85, indicating that the quantum yield of (I) is the second largest. Crystal packing without π-π stack and crystal rigidity in the presence of bulky isopropyl groups probably lead to the enhancement of the fluorescence quantum yield.

### Experimental

1,4,5,8-Tetraisopropylanthracene was prepared according to the method described by Kitamura *et al.* (2007). A mixture of 2,5-diisopropylfuran (1.03 g, 6.78 mmol) and 1,2,4,5-tetrabromobenzene (1.25 g, 3.17 mmol) in dry toluene (40 ml) was cooled to 243 K. To the mixture, 1.6 *M n*-BuLi in hexane (6.0 ml, 9.6 mmol) was added dropwise over 10 min. Then the mixture was warmed up to room temperature over 2 h and stirred at room temperature for additional 18 h. After quenching with water, the aqueous layer was extracted with CHCl_3_. The combined organic layer was washed with brine and dried over Na_2_SO_4_. After evaporation, the residue was subjected to slica-gel chromatography with (2:1)-hexane/CHCl_3_ to afford bis(furan)adduct as a yellow solid (432 mg, 36%). The bis(furan)adduct (432 mg, 1.14 mmol) in EtOH (55 ml) was hydrogenated over 10% Pd/C (85 mg) under atmospheric pressure at room temperature for 3 h. The catalyst was removed by filtration, and the filtrate was evaporated under reduced pressure. To the residue, an ice-cooled solution of (1:5)-conc. HCl/Ac_2_O (6 ml) was added. The mixture was stirred at room temperature for 3 h. After cooling with ice, water was added into the mixture. The resultant mixture was extracted with CHCl_3_, and the extract was washed with aqueous Na_2_CO_3_ and brine, and dried over Na_2_SO_4_. After evaporation of the solvent, column chromatography on silica gel with (2:1)-hexane/CHCl_3_ gave the title compound as a white solid (149 mg, 38%). Recrystallization was performed with Et_2_O to obtain colourless single crystals of the title compound. ^1^H-NMR: *δ* 1.50 (d, J = 6.9 Hz, 24H), 3.87–3.59 (m, 4H), 7.36 (s, 4H), 9.01 (s, 2H); ^13^C-NMR: *δ* 14.06, 23.50, 28.60, 120.60, 129.41, 142.26, 137.03; EIMS: *m*/*z* (%) 346 (100); Elemental analysis for C_26_H_34_: C 90.11, H 9.89%; found: C 89.86, H 9.86%.

### Refinement

All the H atoms were positioned geometrically and refined using a riding model with C–H = 0.94 Å and *U*_iso_(H) = 1.2*U*_eq_(C) for aromatic C—H, C–H = 0.99 Å and *U*_iso_(H) = 1.2*U*_eq_(C) for CH, and C—H = 0.97 Å and *U*_iso_(H) = 1.5*U*_eq_(C) for CH_3_ group.

### Crystal data

**Table:**

C_26_H_34_	*F*(000) = 380
*M**_r_* = 346.53	*D*_x_ = 1.085 Mg m^−^^3^
Monoclinic, *P*2_1_/*c*	Melting point: 488 K
Hall symbol: -P 2ybc	Mo *K*α radiation, λ = 0.71073 Å
*a* = 6.546 (3) Å	Cell parameters from 2745 reflections
*b* = 10.357 (5) Å	θ = 2.4–31.1°
*c* = 15.808 (8) Å	µ = 0.06 mm^−^^1^
β = 98.289 (8)°	*T* = 223 K
*V* = 1060.5 (9) Å^3^	Needle, colourless
*Z* = 2	0.50 × 0.07 × 0.05 mm

### Data collection

**Table:**

Rigaku/MSC Mercury CCD area-detector diffractometer	2817 independent reflections
Radiation source: rotating-anode X-ray tube	1921 reflections with *I* > 2σ(*I*)
graphite	*R*_int_ = 0.043
Detector resolution: 14.7059 pixels mm^-1^	θ_max_ = 29.1°, θ_min_ = 2.4°
φ and ω scans	*h* = −8→8
Absorption correction: numerical (*NUMABS*; Higashi, 2000)	*k* = −14→13
*T*_min_ = 0.991, *T*_max_ = 0.996	*l* = −21→13
9107 measured reflections	

### Refinement

**Table:**

Refinement on *F*^2^	0 restraints
Least-squares matrix: full	Primary atom site location: structure-invariant direct methods
*R*[*F*^2^ > 2σ(*F*^2^)] = 0.063	H-atom parameters constrained
*wR*(*F*^2^) = 0.180	*w* = 1/[σ^2^(*F*_o_^2^) + (0.0945*P*)^2^ + 0.0217*P*] where *P* = (*F*_o_^2^ + 2*F*_c_^2^)/3
*S* = 1.07	(Δ/σ)_max_ < 0.001
2817 reflections	Δρ_max_ = 0.28 e Å^−^^3^
122 parameters	Δρ_min_ = −0.24 e Å^−^^3^

### Special details

**Table:**

Geometry. All s.u.'s (except the s.u. in the dihedral angle between two l.s. planes) are estimated using the full covariance matrix. The cell s.u.'s are taken into account individually in the estimation of s.u.'s in distances, angles and torsion angles; correlations between s.u.'s in cell parameters are only used when they are defined by crystal symmetry. An approximate (isotropic) treatment of cell s.u.'s is used for estimating s.u.'s involving l.s. planes.
Refinement. Refinement of *F*^2^ against ALL reflections. The weighted *R*-factor *wR* and goodness of fit *S* are based on *F*^2^, conventional *R*-factors *R* are based on *F*, with *F* set to zero for negative *F*^2^. The threshold expression of *F*^2^ > 2σ(*F*^2^) is used only for calculating *R*-factors(gt) *etc*. and is not relevant to the choice of reflections for refinement. *R*-factors based on *F*^2^ are statistically about twice as large as those based on *F*, and *R*- factors based on ALL data will be even larger.

### Fractional atomic coordinates and isotropic or equivalent isotropic displacement parameters (Å^2^)

**Table:**

	*x*	*y*	*z*	*U*_iso_*/*U*_eq_	
C1	0.4015 (2)	−0.00637 (14)	0.17133 (9)	0.0272 (3)	
C2	0.2347 (2)	0.06330 (15)	0.18742 (9)	0.0323 (4)	
H2	0.1976	0.0606	0.2427	0.039*	
C3	0.1152 (2)	0.13954 (15)	0.12440 (9)	0.0309 (4)	
H3	0.0041	0.1874	0.1397	0.037*	
C4	0.1553 (2)	0.14606 (13)	0.04219 (9)	0.0254 (3)	
C5	0.3279 (2)	0.07195 (13)	0.02069 (9)	0.0245 (3)	
C6	0.4533 (2)	−0.00120 (13)	0.08561 (8)	0.0245 (3)	
C7	0.6226 (2)	−0.06900 (13)	0.06259 (9)	0.0263 (3)	
H7	0.7074	−0.1149	0.1054	0.032*	
C8	0.5294 (2)	−0.08656 (16)	0.24020 (9)	0.0336 (4)	
H8	0.5737	−0.1653	0.2123	0.04*	
C9	0.7238 (3)	−0.0145 (2)	0.27785 (12)	0.0551 (5)	
H9A	0.6861	0.0624	0.307	0.083*	
H9B	0.801	0.0099	0.2324	0.083*	
H9C	0.8083	−0.0699	0.3182	0.083*	
C10	0.4106 (3)	−0.1305 (2)	0.31140 (11)	0.0523 (5)	
H10A	0.4928	−0.1925	0.3475	0.078*	
H10B	0.2817	−0.1703	0.2865	0.078*	
H10C	0.3817	−0.0565	0.3454	0.078*	
C11	0.0299 (2)	0.23022 (14)	−0.02482 (9)	0.0293 (3)	
H11	−0.0029	0.1782	−0.0776	0.035*	
C12	−0.1738 (2)	0.27689 (16)	0.00091 (11)	0.0363 (4)	
H12A	−0.2497	0.2037	0.0188	0.054*	
H12B	−0.2552	0.3188	−0.0475	0.054*	
H12C	−0.1459	0.3377	0.0478	0.054*	
C13	0.1551 (3)	0.34794 (16)	−0.04622 (11)	0.0412 (4)	
H13A	0.0758	0.3967	−0.092	0.062*	
H13B	0.2832	0.3193	−0.0642	0.062*	
H13C	0.1855	0.4022	0.004	0.062*	

### Atomic displacement parameters (Å^2^)

**Table:**

	*U*^11^	*U*^22^	*U*^33^	*U*^12^	*U*^13^	*U*^23^
C1	0.0275 (7)	0.0305 (7)	0.0245 (7)	−0.0017 (6)	0.0070 (6)	0.0001 (6)
C2	0.0338 (8)	0.0401 (9)	0.0251 (7)	0.0018 (7)	0.0115 (6)	0.0008 (6)
C3	0.0279 (7)	0.0345 (8)	0.0321 (8)	0.0042 (6)	0.0107 (6)	−0.0020 (6)
C4	0.0240 (7)	0.0257 (7)	0.0271 (7)	−0.0013 (6)	0.0053 (6)	−0.0018 (6)
C5	0.0225 (7)	0.0263 (7)	0.0252 (7)	−0.0018 (6)	0.0056 (6)	−0.0028 (5)
C6	0.0245 (7)	0.0272 (7)	0.0227 (7)	−0.0009 (6)	0.0061 (6)	−0.0011 (6)
C7	0.0257 (7)	0.0294 (7)	0.0238 (6)	0.0020 (6)	0.0038 (6)	0.0007 (6)
C8	0.0351 (9)	0.0418 (9)	0.0258 (7)	0.0053 (7)	0.0106 (6)	0.0038 (6)
C9	0.0487 (11)	0.0736 (14)	0.0393 (10)	−0.0036 (10)	−0.0065 (9)	0.0062 (10)
C10	0.0560 (12)	0.0639 (13)	0.0418 (10)	0.0165 (10)	0.0230 (9)	0.0225 (9)
C11	0.0286 (7)	0.0305 (8)	0.0292 (7)	0.0049 (6)	0.0054 (6)	−0.0009 (6)
C12	0.0313 (8)	0.0381 (9)	0.0401 (9)	0.0070 (7)	0.0069 (7)	0.0013 (7)
C13	0.0412 (9)	0.0374 (9)	0.0462 (9)	0.0056 (8)	0.0107 (8)	0.0105 (8)

### Geometric parameters (Å, °)

**Table:**

C1—C2	1.362 (2)	C8—H8	0.99
C1—C6	1.4448 (18)	C9—H9A	0.97
C1—C8	1.521 (2)	C9—H9B	0.97
C2—C3	1.416 (2)	C9—H9C	0.97
C2—H2	0.94	C10—H10A	0.97
C3—C4	1.364 (2)	C10—H10B	0.97
C3—H3	0.94	C10—H10C	0.97
C4—C5	1.4463 (19)	C11—C12	1.528 (2)
C4—C11	1.518 (2)	C11—C13	1.534 (2)
C5—C7^i^	1.4009 (19)	C11—H11	0.99
C5—C6	1.435 (2)	C12—H12A	0.97
C6—C7	1.4033 (19)	C12—H12B	0.97
C7—C5^i^	1.4009 (19)	C12—H12C	0.97
C7—H7	0.94	C13—H13A	0.97
C8—C9	1.520 (3)	C13—H13B	0.97
C8—C10	1.527 (2)	C13—H13C	0.97
			
C2—C1—C6	117.28 (13)	H9A—C9—H9B	109.5
C2—C1—C8	121.93 (12)	C8—C9—H9C	109.5
C6—C1—C8	120.79 (12)	H9A—C9—H9C	109.5
C1—C2—C3	122.72 (12)	H9B—C9—H9C	109.5
C1—C2—H2	118.6	C8—C10—H10A	109.5
C3—C2—H2	118.6	C8—C10—H10B	109.5
C4—C3—C2	122.35 (13)	H10A—C10—H10B	109.5
C4—C3—H3	118.8	C8—C10—H10C	109.5
C2—C3—H3	118.8	H10A—C10—H10C	109.5
C3—C4—C5	117.50 (13)	H10B—C10—H10C	109.5
C3—C4—C11	122.28 (13)	C4—C11—C12	113.63 (12)
C5—C4—C11	120.19 (12)	C4—C11—C13	110.97 (13)
C7^i^—C5—C6	118.33 (12)	C12—C11—C13	108.77 (13)
C7^i^—C5—C4	121.79 (13)	C4—C11—H11	107.7
C6—C5—C4	119.88 (12)	C12—C11—H11	107.7
C7—C6—C5	118.08 (12)	C13—C11—H11	107.7
C7—C6—C1	121.72 (13)	C11—C12—H12A	109.5
C5—C6—C1	120.19 (12)	C11—C12—H12B	109.5
C5^i^—C7—C6	123.56 (13)	H12A—C12—H12B	109.5
C5^i^—C7—H7	118.2	C11—C12—H12C	109.5
C6—C7—H7	118.2	H12A—C12—H12C	109.5
C9—C8—C1	110.83 (14)	H12B—C12—H12C	109.5
C9—C8—C10	110.13 (15)	C11—C13—H13A	109.5
C1—C8—C10	113.81 (13)	C11—C13—H13B	109.5
C9—C8—H8	107.3	H13A—C13—H13B	109.5
C1—C8—H8	107.3	C11—C13—H13C	109.5
C10—C8—H8	107.3	H13A—C13—H13C	109.5
C8—C9—H9A	109.5	H13B—C13—H13C	109.5
C8—C9—H9B	109.5		
			
C6—C1—C2—C3	−0.6 (2)	C8—C1—C6—C7	−0.2 (2)
C8—C1—C2—C3	179.32 (14)	C2—C1—C6—C5	−1.8 (2)
C1—C2—C3—C4	1.7 (2)	C8—C1—C6—C5	178.27 (13)
C2—C3—C4—C5	−0.4 (2)	C5—C6—C7—C5^i^	−1.8 (2)
C2—C3—C4—C11	−178.60 (14)	C1—C6—C7—C5^i^	176.64 (13)
C3—C4—C5—C7^i^	177.89 (13)	C2—C1—C8—C9	−99.18 (17)
C11—C4—C5—C7^i^	−3.8 (2)	C6—C1—C8—C9	80.72 (18)
C3—C4—C5—C6	−2.0 (2)	C2—C1—C8—C10	25.6 (2)
C11—C4—C5—C6	176.24 (12)	C6—C1—C8—C10	−154.51 (15)
C7^i^—C5—C6—C7	1.7 (2)	C3—C4—C11—C12	−15.2 (2)
C4—C5—C6—C7	−178.32 (12)	C5—C4—C11—C12	166.61 (13)
C7^i^—C5—C6—C1	−176.76 (13)	C3—C4—C11—C13	107.76 (16)
C4—C5—C6—C1	3.2 (2)	C5—C4—C11—C13	−70.44 (17)
C2—C1—C6—C7	179.72 (14)		

Symmetry codes: (i) −*x*+1, −*y*, −*z*.
